# Phytoene Accumulation in the Novel Microalga *Chlorococcum* sp. Using the Pigment Synthesis Inhibitor Fluridone

**DOI:** 10.3390/md17030187

**Published:** 2019-03-22

**Authors:** Kelly Laje, Mark Seger, Barry Dungan, Peter Cooke, Juergen Polle, F. Omar Holguin

**Affiliations:** 1 Department of Plant and Environmental Sciences, New Mexico State University, Las Cruces, NM 88003, USA; klaje@nmsu.edu (K.L.); bdungan@nmsu.edu (B.D.); 2 AzCATI, School of Sustainable Engineering and the Built Environment, Arizona State University, Mesa, AZ 85212, USA; mseger1@asu.edu; 3 Core University Research Resources Laboratory, New Mexico State Univesrity, Las Cruces, NM 88003, USA; phcooke@nmsu.edu; 4 Department of Biology, Brooklyn College of the City University of New York, Brooklyn, NY 11210, USA; JPolle@brooklyn.cuny.edu; 5 The Graduate Center of the City University of New York, 365 Fifth Avenue, New York, NY 10016, USA

**Keywords:** phytoene, carotenoids, antioxidants, fluridone, microalgae, cosmeceuticals

## Abstract

Carotenoids are lipophilic pigments found in plants and algae, as well as some bacteria, archaea, and fungi that serve two functions—(1) as light harvesting molecules—primary carotenoids, and (2) as antioxidants, acting against reactive oxygen species–secondary carotenoids. Because of their strong antioxidant properties, they are also valuable for the development of anti-aging and photo-protective cosmetic applications. Of particular interest is the carotenoid phytoene, for its colorless and UV absorption characteristics. In this study, we targeted a reduction of phytoene desaturase (PDS) activity with the pigment-inhibiting herbicide 1-methyl-3-phenyl-5-[3-(trifluoromethyl)phenyl]pyridin-4-one (fluridone), which leads to the over-accumulation of phytoene in the recently characterized microalgal strain *Chlorococcum* sp. (UTEX B 3056). After post-incubation with fluridone, phytoene levels were measured at ~33 ug/mg cell tissue, as opposed to non-detectable levels in control cultures. Hence, the novel microalga *Chlorococcum* sp. is a viable candidate for the production of the high-value carotenoid phytoene and subsequent applications in cosmeceuticals, as well as more obvious nutraceutical and pharmaceutical applications.

## 1. Introduction

Microalgae are known to be potential sources of natural products, abundant and versatile in their activity and applications. Of particular importance are the lipophilic pigments, carotenoids. Commonly used in the food and nutraceuticals industry as colorants and dietary supplements, carotenoids have received growing popularity in cosmetics in large part, due to their antioxidant properties [1,2,3,4]. Synthesized in chloroplasts, carotenoids are a part of the photosynthetic complex (primary carotenoids), absorbing light in the 400–500 nm range, and also acting as a defense system in the presence of high light intensity or oxidative stress (secondary carotenoids) [5,6,7]. Secondary carotenoids act to quench singlet oxygen species and trap peroxyl radicals, protecting the cell from lipid peroxidation in both plants and animals [8,9,10,11,12]. Studies have shown that carotenoids also possess anti-inflammatory and immunomodulatory effects in animal tissues [8,13,14]. These qualities have made secondary carotenoids the subject of intense research surrounding anti-cancer therapies and heart disease, among others [8,15,16].

Carotenoids are either pure hydrocarbon molecules (carotenes) or oxygenated derivatives of carotenes (xanthophylls), all of which are comprised of a 40 carbon atom chain. One conjugated double bond is added with every carotenoid produced downstream of phytoene, in the synthetic chain, having a direct impact on the antioxidant strength of the molecule [8,16,17]. Thus, carotenoids are of particular importance for their potential as a natural source of antioxidants. The first carotenoid in the terpenoid pathway is phytoene; a symmetric, linear branched carotenoid with nine conjugated double bonds, produced from two C20 molecules of geranylgeranyl pyrophosphate (GGPP), and catalyzed by the enzyme phytoene synthase (PSY) [17,18]. In plants and green algae, phytoene progresses to phytofluene and ζ-carotene via phytoene desaturase (PDS). Subsequently, the carotenoid biosynthesis pathway proceeds to the carotenes–lycopene, and by ring introduction, to α-carotene and β-carotene; and then further to the xanthophylls–lutein (from α-carotene) and zeaxanthin (from β-carotene), respectively [5,16,18]. Secondary carotenoids are synthesized and accumulated during unfavorable growth conditions, such as high irradiance and/or nutrient deprivation, in which carotenoids contribute to cell protection (e.g., light absorption at a photosynthetic range beyond the capacity of chlorophyll) [19,20]. Depending on the species of alga, these secondary carotenoids may accumulate in carotene globules within the chloroplast [21,22] or in oil bodies in the cytosol, as seen during astaxanthin production in *Haematococcus pluvialis* [23,24].

Phytoene absorbs light in the ultraviolet range, and is colorless in nature; qualities that add to its value in cosmetic formulation as a skin protectant [13,25]. Current sources of phytoene come from tomato extract [26,27] and the carotenogenic microalga *Dunaliella bardawil* [28,29,30]. However, phytoene is difficult to accumulate in large quantities because, as a precursor molecule, it is used in the downstream synthesis of other primary and secondary carotenoids [18]. Phytoene levels in tomato (ripe) and *D. bardawil* (stress-induced) range from ~2–9 µg/g dry weight [31,32,33], and 8% (80 mg/g) [28], respectively.

Previous studies successfully induced the over accumulation of phytoene through the use of pigment synthesis inhibiting herbicides [29,31,32,33]. These bleaching herbicides target the enzyme phytoene desaturase (PDS), responsible for the downstream production of carotenoids past the metabolic step of phytoene production [34]. The inability to synthesize carotenoids that are essential for structure and function of photosynthetic complexes results in chlorophyll degradation, and ultimately, plant cell death [10,35,36,37]. At non-lethal doses, effective inhibition of PDS leads to the over-accumulation of phytoene [23,29,31,32,35,38]. This has been demonstrated in the microalgae *D. bardawil* and *H. pluvialis*, in which phytoene accumulation increased sharply as a result of exposure to bleaching herbicides [29,31,32,33]. *Chlamydomonas reinhardtii*, *H. pluvialis*, and the cyanobacteria *Synechococcus* have been studied extensively for norflurazon (5-amino-4-chloro-2-[3-(trifluoromethyl)phenyl]pyridazin-3-one) and fluridone (1-methyl-3-phenyl-5-[3-(trifluoromethyl)phenyl]pyridine-4-one) resistance mechanisms and mutagenesis, as well as herbicide inhibition activity [33,34,38,39,40,41].

In this study, our objective was to over-accumulate the carotenoid phytoene in a novel strain of green microalga, *Chlorococcum* sp. (UTEX B 3056), a fresh-water algae that closely resembles *C. reinhardtii* [42,43,44]. *Chlorococcum* exists as a unicellular, spheroidal organism, in either a vegetative (non-motile) or a zoospore (bi-flagellate) state [42,43]. We chose to study this strain of *Chlorococcum* sp. because it is highly carotenogenic, fast-growing, produces large quantities of biomass, and can be cultivated outdoors in raceway-type ponds [42,45]. We optimized the concentration of fluridone to facilitate the accumulation of phytoene without inducing bleaching and cell death. Furthermore, we characterized the effects of phytoene accumulation on the carotenoid and fatty acid (FA) profiles of cell extracts.

## 2. Results

### 2.1. Strain Identification & Morphology

Briefly, sequencing of the 18S rDNA confirmed previous characterization of the ITS2 region by Neofotis, et al., linking this alga to *Chlorococcum* sp. (Supplementary Figure S1) [42]. Neofotis, et al. pointed out that query coverage is low with this species and that unambiguous identification of this group at the species level, even with use of the ITS2 marker, is not definitive due to a lack of sequence availability in the public databases [42]. Morphological characterization via bright field and scanning electron microscopy agreed with molecular taxonomy; these images are provided in supplementary materials (Supplementary Figure S2).

### 2.2. Microplate Bioassays

**Figure 1:**
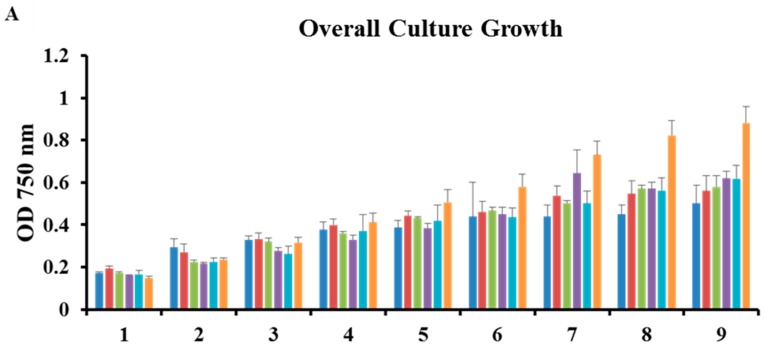

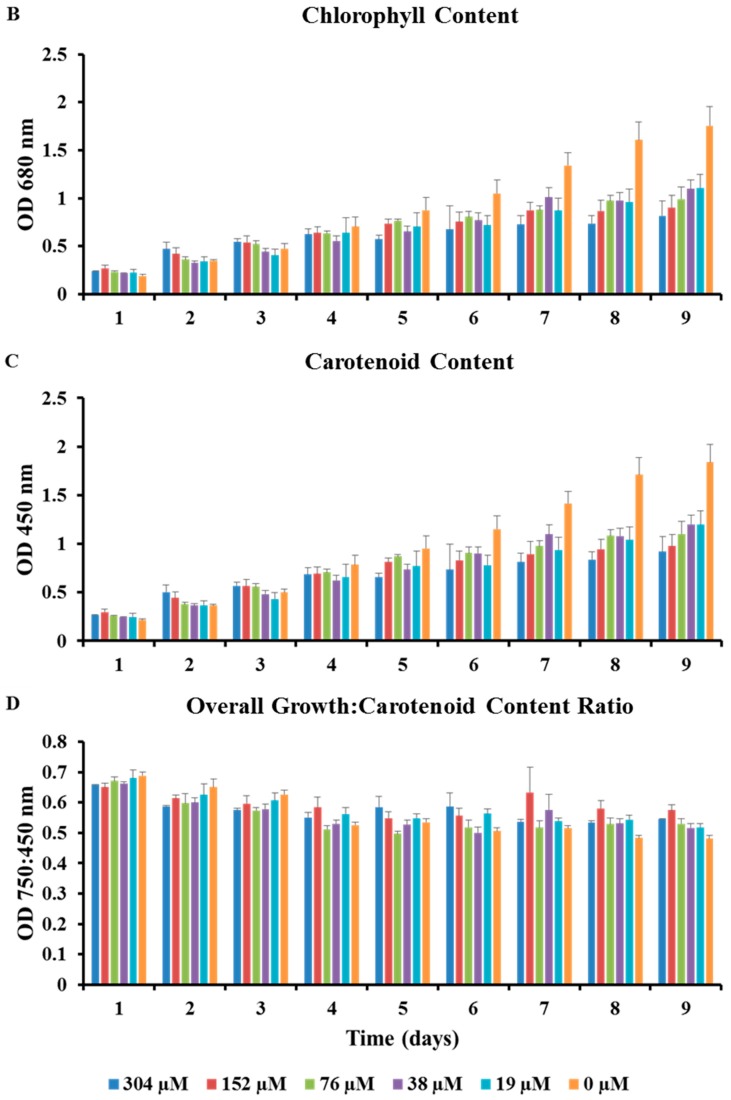
Micro-plate bioassay growth charts of *Chlorococcum* sp. over a series of fluridone treatments (**A**) Overall Culture Growth (750 nm); (**B**) Chlorophyll Content (680 nm); (**C**) Carotenoid Content (450 nm); (**D**) Overall Growth:Carotenoid Content Ratio used to determine optimal fluridone concentration (750:450 nm). * n = 4 for all samples; excluding day 6–304 µM, where n = 3. Asterisks indicate statistical significance in panels A–C. Significance in panel D not applicable.

*Chlorococcum* sp. growth was analyzed in the presence of fluridone at serial concentrations via UV spectrophotometric readings at the following wavelengths: 750 nm (overall growth), 680 nm (chlorophyll content), 450 nm (carotenoid content) (Figure 1) [7]. Note that cultures were started at an OD of 0.1 (day zero), and growth monitoring began the following day (day 1) (Figure 1). The overall growth and chlorophyll/carotenoid content of the cultures was significantly impacted at all concentrations of fluridone; thus, there appears to be no difference between the OD at each wavelength amongst the trends (panels A–C, Figure 1) [7]. The graph representing 750/450 nm showed highest growth/lowest carotenoid content in the 152 µM concentration. Upon experimental scale-up, we chose to treat cultures with the two highest doses, 152 µM and 304 µM, to observe the effects of the optimal concentration (152 µM), as well as the effects of a stronger dose (304 µM), on culture growth and phytoene accumulation (panel D, Figure 1). Although 152 µM does not appear to be significantly different between early and later time points in the 750/450 nm ratio, this is likely due to cell death and pigment inhibition over the course of the treatment (panel D, Figure 1). A two way repeated measures ANOVA, using the Holm-Sidak method, was performed to measure the significance of growth period and concentration. Herbicidal effects were dosage dependent, with a statistically significant interaction between day and concentration (*P* ≤ 0.001). Asterisks denote treatments in which significance was observed (panels A–C, Figure 1). However, it should be noted that 152 µM (panel A, Figure 1) and 38 µM (panels A–C, Figure 1) treatments have a *p* value of 0.007 and ~0.02, respectively, on day seven. Significance is not noted in panel D (Figure 1), as there was no statistical significance observed between treatments within a given day, unlike for panels A–C.

### 2.3. Phytoene Quantification

Results in Panel B, Figure 1 (chlorophyll absorbance) indicate that algal growth begins to slow after day 4, and statistically significant differences in growth between treated and untreated cultures (panels A–C, Figure 1) are observed at day 7 and beyond. The statistical significance that occurs at days 7–9 indicates treated cultures were not growing as optimally as the control. Hence, we chose to harvest cell tissue for phytoene analysis when cultures were in optimal growth (day 4). Carotenoid extraction and subsequent HPLC analysis of 25 mL cultures *Chlorococcum* sp. incubated with 152 µM and 304 µM fluridone revealed the accumulation of phytoene at approximately 33 μg/mg of phytoene per dry cell weight when harvested on day four, as well as a reduction in downstream carotenoid production at both concentrations (Table 1). At the fourth-day harvest, there was no notable increase of phytoene accumulation when increasing the fluridone dose from 152 µM and 304 µM. (Table 1). Differences in phytoene levels became apparent when cell tissue was harvested after a nine-day incubation period. Phytoene quantification of this tissue revealed a reduction in the amount of phytoene accumulated in cultures treated with 304 µM as compared to 152 µM fluridone, at only 4.6 µg/mg, versus 14.6 µg/mg, respectively (Table 1). Carotenoid content at both harvest periods was reduced in fluridone-treated cultures by approximately half that seen in non-treated cultures ~40 µg/mg (treated cultures) vs. 70 µg/mg (controls), and ~70 µg/mg (treated cultures) vs. 145 µg/mg (controls), at the four-day and nine-day harvest, respectively (Table 1). Final carotenoid levels were within a standard deviation between concentrations.

**Figure 2:**
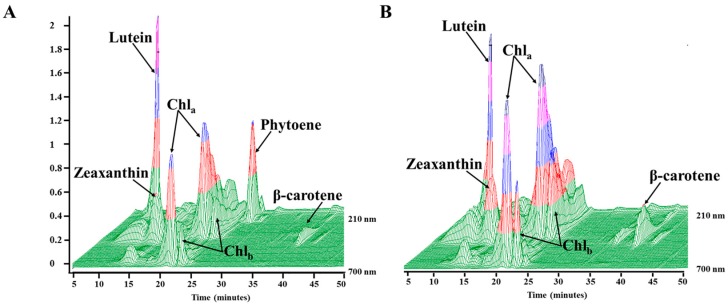
HPLC 3D chromatograms of cellular extracts from *Chlorococcum* sp., day 4 harvest (**A**) exposed to fluridone (152 µM). Note that phytoene was detected eluting at ~27 min. (**B**) the absence of phytoene without the addition of fluridone. Lutein, zeaxanthin, β-carotene and chlorophyll *a/b* are also denoted.

Panel A, Figure 2 shows phytoene eluting at approximately twenty-seven minutes, absorbing at 284 nm in cultures that had been harvested on day 4 of treatment with 152 µM fluridone, and a relatively low amount of carotenoid production is observed. Chromatograms for controls (cultures without fluridone) contained no peak for phytoene (panel B, Figure 2) and exhibited downstream carotenoid products (i.e., lutein, zeaxanthin, and β-carotene).

### 2.4. Fatty Acid Analysis

**Figure 3:**
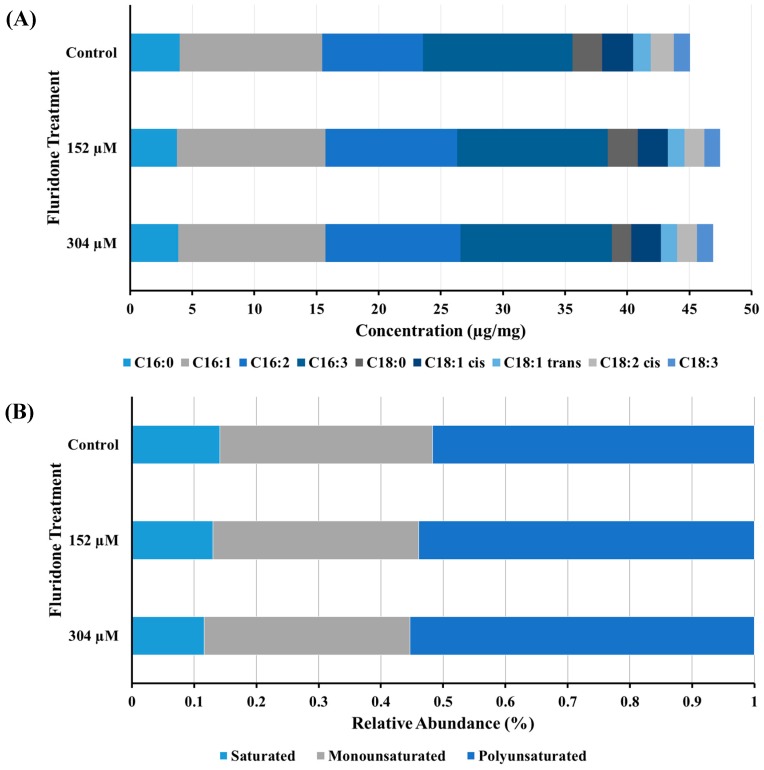
Fatty acid methyl ester (FAME) analysis of cellular extracts obtained on the day 4 harvest period from *Chlorococcum* sp. (**A**) total FA content, (**B**) relative abundance of saturated, monounsaturated, and polyunsaturated FAs.

The fatty acid profile of cellular extracts obtained on day 4 from *Chlorococcum* sp. were analyzed for the observation of any potential downstream effects on fatty acid desaturase enzymes, in which previous studies have shown herbicides with this mode of action have exhibited inhibitory effects [23]. The FAs C16:0 and C16:3 remained relatively conserved within concentrations and controls, comparatively speaking. (panel A, Figure 3). However, the mono and poly-unsaturated FAs showed a slight increase in the presence of fluridone, from ~11 µg/mg (controls) to ~12 µg/mg (+ fluridone), and from ~8 µg/mg (controls) to ~11 µg/mg (+ fluridone), in C16:1 and C16:2, respectively (panels A & B, Figure 3). The increase in abundance of the aforementioned FAs was similar in both fluridone treatments (panels A & B, Figure 3). The abundance of the mono-unsaturated and poly-unsaturated FAs C18:1 cis/trans, C18:2 cis, and C18:3 in cultures incubated with 152 µM and 304 µM fluridone were not significantly different from that of the control cultures or between concentrations; < 0.5 µg/mg difference (panel A, Figure 3). C18:0 concentration increased slightly in cultures incubated at 152 µM: from ~1.5 µg/mg (304 µM), to ~2.5 µg/mg (152 µM) (panel A, Figure 3). A two-way analysis of variance (ANOVA), using the Holm-Sidak method, was performed to determine any significance between FA levels, fluridone treatment, and treatment concentration (152 µM vs. 304 µM). Statistical significance has been noted for FA abundance between herbicide treatments and controls. However, statistical significance was not observed when comparing the two treatment concentrations. In other words, we did not see a significant change in the effect of 152 µM over 304 µM and the resulting FA abundance, overall. It should be noted, though, that C18:0 abundance was significantly different between the two concentrations, as an exception to the former statement. *P* ≤ 0.001. Note: * n = 3 for all samples; excluding C16:2, where n = 2. Total FAME concentrations are also outlined in the supplementary material, Supplementary Table S2 (S3).

### 2.5. Intracellular Oil Body Visualization

**Figure 4:**
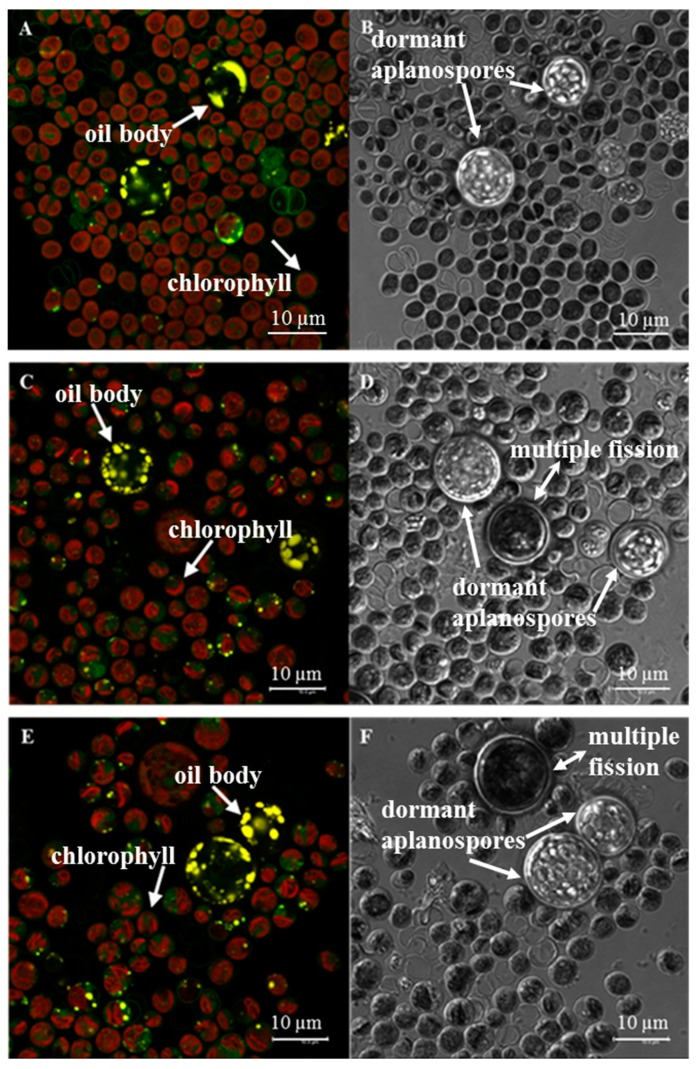
Confocal fluorescence images (left); corresponding DIC images (right) of *Chlorococcum* sp.—panels (**A**,**B**) without fluridone, (**C**,**D**) with 152 µM fluridone, (**E**,**F**) with 304 µM fluridone. Arrows in panels (**A**,**C**,**E**) point either to cells with oil bodies (yellow fluorescence) or chlorophyll (red fluorescence); arrows in panels (**B**,**D**,**F**) point to dormant aplanospores (containing oil bodies), and multiple fission cells (double-headed arrows).

Confocal fluorescence microscopy indicated non-uniformity/streaking of the chlorophyll (red fluorescence) in fluridone treated cultures, as opposed to control cultures, which showed fuller/more uniform chlorophyll fluorescence throughout the cell (panels C & E, Figure 4). This might indicate chloroplastic degradation in cultures incubated with fluridone. We also observed a minor increase in the number of oil bodies formed in cultures treated with both concentrations of fluridone, characterized by yellow fluorescent droplets within zoospores (smaller cells) and dormant aplanospores (larger cells) (panels C & E, Figure 4). Note that the dormant aplanospores are large cysts containing oil bodies that fluoresce yellow when observed microscopically [46]; whereas, the large cells that did not fluoresce yellow are simply cells undergoing multiple fission–a process whereby a mitotic cell gives rise to several daughter cells [47]. Dormant aplanospores and cells undergoing multiple fission are labeled in the differential interference contrast images (DIC)-panels B, D, & F, Figure 4, as the corresponding images to panels A, C, & E, Figure 4. DIC images were taken to better define intracellular bodies (panels B, D, & F, Figure 4). Further study is needed to elucidate the intracellular location of phytoene, and whether it is accumulated in oil bodies or elsewhere within the cells.

## 3. Discussion

### 3.1. Strain Identification & Microplate Bioassays

*Chlorococcum* sp. identity was confirmed molecularly (DNA) and morphologically [41]. Microplate inhibition bioassays were used to determine appropriate herbicide concentration for optimal phytoene desaturase inhibition, adapted from Franz, et al. [48]. Phytoene absorbs at approximately 280 nm, whereas carotenoids downstream of phytoene absorb in the 400 nm to 500 nm range. Therefore, cultures that showed highest overall biomass accumulation as determined by measuring the optical density at 750 nm, coupled with lowest carotenoid development, measured at 450 nm, were indicative of the optimal herbicide concentration at which greatest PDS inhibition was achieved without cell death. As such, 152 µM fluridone was chosen as the optimal concentration to achieve carotenoid inhibition without severely limiting growth (panel D, Figure 1). Cultures were also treated with 304 µM fluridone upon experimental scale-up to observe any notable differences between the concentrations, of which no significant differences in the overall accumulation of phytoene were seen (Table 1).

Similar studies found that the pigment synthesis inhibitor norflurazon caused an 80% decrease of the secondary carotenoid β-carotene in the alga *D. bardawil* at a concentration of 0.1 μM, with concurrent accumulation of phytoene [29]. Other studies have found concentrations of norflurazon ranging from 0.02 μM to 0.3 μM and 100 μM to be effective concentrations for PDS inhibition in the algae *H. pluvialis* and *D. bardawil*, respectively, with substantial accumulation of phytoene in both species [23,31,32]. However, unlike similar studies where cultures were treated with pigment synthesis inhibitors during a carotenogenic state [28], we have chosen to treat during exponential growth phase for the purpose of achieving maximum biomass during phytoene accumulation. A study into the inhibitory effects of fluridone on *E. coli* expressed PDS from the cyanobacterium *Synechococcus* (PCC 7492), as well as purified *Synechococcus* PDS, revealed a concentration of 0.3 μM and 3.5 μM to cause 50% inhibition of carotenoid production, respectively [38]. Chalifour, et al. discovered that a range of temperatures influences the inhibitory effects of the herbicides norflurazon and fluridone in the model alga *C. reinhardtii* [35]. It was found that 1.25 μM fluridone had the greatest impact on secondary carotenoid formation at a temperature of 25 °C; whereas, secondary carotenoid formation was affected to a lesser extent at lower temperatures [35].

### 3.2. Phytoene Quantification

The insignificant increase of phytoene accumulation between 304 µM and 152 µM concentrations of fluridone at day four (Table 1) was likely due to the inhibition of downstream carotenoid synthesis, and therefore, the inability to maintain the photosynthetic complex at a fluridone concentration greater than 152 µM, resulting in increased cell death. This tentative conclusion is supported in previous studies where photosynthetic complexes I and II, particularly system II, are negatively impacted and experience some form of inhibition in the presence of pigment-synthesis inhibitors—fluridone and/or norflurazon [28,49,50]. Therefore, when carotenoid synthesis is inhibited, the photosynthetic complex degrades [10,35,36,37]. Phytoene levels were further reduced at the nine-day time point. Therefore, we suspect that the strongest concentration of fluridone applied for this study (304 µM), in conjunction with a longer incubation period (9 days), leads to increased cell death and an overall reduction in phytoene accumulation/carotenoid development. For future study, it would be wise to measure phytoene content, cell viability, and photosynthetic inhibition using Fv/Fm measurements, on a daily basis to draw better conclusions that may refute or support these statements.

Decreased carotenoid production coupled with a significant peak for phytoene, as seen in panel A, Figure 2, is a result of successful PDS inhibition by the herbicide fluridone. Results observed in Figure 2 are consistent with previous studies where inhibition of PDS by the pigment synthesis inhibitors fluridone and norflurazon resulted in the over-accumulation of phytoene. One study showed that phytoene constituted 60% of total carotenoid content in norflurazon treated *H. pluvialis* [23,32]. Large amounts of phytoene accumulation in the alga *D. bardawil* have been reported in two separate studies through the use of the inhibitor norflurazon [29,31]. Norflurazon has been a popular choice for PDS inhibition; thus, there is a need for further research into the inhibition capabilities of fluridone for the purpose of carotenoid regulation and potential phytoene accumulation.

### 3.3. FAME Analysis & Confocal Fluorescence Microscopy

We speculate that the increase in the unsaturated FAs C16:1 and C16:2 may be due to oil body formation as a response to induced stress (panel A, Figure 3). The literature describes inhibition of FA desaturase enzyme activity by pigment synthesis inhibitors, resulting in decreased levels of lipids, especially the mono-unsaturated and poly-unsaturated FAs [23,35]. The observed increase in C16:1 and C16:2 FAs in this study suggests that the applied concentrations of fluridone did not result in inhibition of FA desaturases, however was likely an effect of lipid remodeling during triacylglycerol synthesis and oil body formation. However, research has shown that, in the alga *C. reinhardtii*, temperature and inhibitor dosage play a large role in the amount of FA desaturase inhibition when exposed to a pigment synthesis inhibitor [35]. Zhekisheva et al. observed the simultaneous decrease in total FA and oleic FA content with increasing concentrations of norflurazon [23]. Notably, C18:0 abundance is markedly and significantly decreased in cultures treated with 304 µM. This same phenomenon was not observed in cultures treated with 152 µM, nor were there any statistically significant differences in the abundance of FAs downstream of C18:0 when compared to controls (no treatment), or in either treatment concentration. As previously mentioned, we suspect these observations are the result of cell death at higher concentrations of fluridone. As in the case of phytoene concentration, future studies should include daily FAME analysis and live cell counts to better understand the effects of various concentrations of fluridone on the metabolic profile and overall lifespan of *Chlorococcum* sp.

We further investigated oil body formation and phytoene accumulation via confocal fluorescence microscopy to determine a relationship between the two, if any. It has been reported that secondary carotenoid formation, specifically β-carotene and astaxanthin, and the accumulation/storage thereof, is directly related to overall FA content and oil body formation [23,29,35,51,52]. The increase in oil bodies within dormant aplanospores seen in fluorescence images, as well as the slight increase in C16:1 and C16:2 FAs in cultures treated with fluridone, may be explained as either a stress response to the herbicide, and/or an accumulation site for phytoene, as is seen in *H. pluvialis* for the storage of astaxanthin [23,24,46]. Therefore, FAME and fluorescence microscopy results should be considered together.

Fluorescence microscopy provided further insights into the effects of fluridone on the photosynthetic apparatus. We speculate that the chloroplastic bifurcation observed in panels C & E, Figure 4 occurs as a result of carotenoid inhibition. Chalifour et al. 2014 found a decrease in chlorophyll *a/b* content and photosynthetic capacity of *C. reinhardtii* when exposed to norflurazon and fluridone. This is not surprising, as carotenoid inhibition with bleaching herbicides results in a loss of the ability to maintain and protect the photosynthetic complex. Therefore, when carotenoid synthesis is inhibited, the photosynthetic complex degrades [10,35,36,37].

Although informative, the precise location of phytoene cannot be determined, conclusively, using the methods discussed above. Further investigation utilizing spatial and molecular signature tools, such as Raman spectroscopy, are needed to better understand the site and mechanism of phytoene accumulation.

## 4. Conclusions

The pigment synthesis inhibitor fluridone was effective in the over-accumulation of phytoene in the novel microalga *Chlorococcum* sp. Our observations indicate that higher concentrations of the inhibitor fluridone do not result in an increase of phytoene; therefore, lower concentrations of the inhibitor may be a more efficient and effective choice for producers utilizing this method. However, PDS mutagenesis for enhanced phytoene production may be even more effective than the use of pigment synthesis inhibitors. Thus, genomic sequencing of *Chlorococcum* sp., followed by bioinformatics research, is necessary to understand PDS expression in this strain, and how targeted mutagenesis may proceed from those findings. Based on these conclusions, *Chlorococcum* sp. should be considered a valuable candidate in the production of high-value carotenoids for cosmetics, and other biomedical studies for which carotenoids are relevant.

## 5. Materials and Methods

### 5.1. Cultivation

Cultivation of *Chlorococcum* sp. (UTEX B 3056) was performed in sterile BD Falcon™ Tissue Culture Flasks with vented caps from BD Biosciences (Erembodegem, BE), grown in BG11 media at 24 °C in an incubator with 1% CO_2_ and atmosphere illuminated with cool white fluorescent lamps (22μE per s^−1^m^−2^). The composition of the liquid medium is as described by the UTEX Culture Collection of Algae (The University of Texas at Austin, Austin, TX, USA).

### 5.2. Strain Identification

Molecular characterization and identification were performed on the genomic DNA extracted from *Chlorococcum* sp. DOE 0101 using the PowerSoil DNA Isolation Kit (Mo Bio Laboratories; Carlsbad, CA, USA). Regions of the 18S rDNA and the RuBisCo Large subunit were amplified from genomic DNA by polymerase chain reaction (PCR) using universal primer sets 18S rDNA (Forward—GTCAGAGGTGAAATTCTTGGATTTA, Reverse—AAGGGCAGGGACGTAATCAACG) and the RuBisCo Large subunit (Forward—AACCTTTCATGCGTTGGAGAGA, Reverse—CCTGCATGAATACCACCAGAAGC) and the GoTaq^®^ colorless master mix (Promega; Madison, WI, USA) according to the manufacturer’s instructions. The PCR reactions were performed on a Mastercycler gradient machine (Eppendorf, Wesbury, NY, USA). The PCR program consisted of an initial denaturation/activation step at 95 °C (3 min), 35 cycles of amplification [DNA denaturation step at 95 °C (30 s), followed by an annealing step at 57 °C (30 s) and an elongation step at 72 °C (45 s)], and a final elongation step at 57 °C (10 min). Amplicons were checked for size verification and specificity by gel electrophoresis on a 1% agarose gel. The amplicons were purified from gels using an UltraClean GelSpin^®^ DNA extraction kit (Mo Bio Laboratories; Carlsbad, CA, USA) for subsequent forward and reverse sequencing (Sanger; ABI 3730 DNA analyzer) at the Functional Biosciences laboratory (Madison, WI, USA). Sequence data was analyzed and assembled using Geneious^®^ (V6.1.4; Biomatters Inc., Newark, NJ, USA). The consensus sequences were subjected to standard nucleotide similarity searches via BLASTn [53] against the NCBI non-redundant database using standard parameters to determine their identities and assess their similarities to those in NCBI GenBank.

### 5.3. Microscopy

Concentrated suspensions of three series of preparation: (1) fresh, (2) 2.5% glutaraldehdye-fixed (Electron Microscopy Sciences, Hatfield, PA, USA) and (3) fixed-Nile Red (Sigma-Aldrich Corp., St. Louis, MO, USA) (5 micrograms/mL) treated cells were deposited onto the coverslip areas in glass bottom microwell dishes (MatTek Corp., Ashland, MA, USA) and examined by confocal microscopy using a model TCS SP5 system coupled to a DMI 6000 inverted microscope equipped with a 100× objective lens (Leica Microsystems, Exton, PA, USA) in the x,y,z imaging mode and fluorescence scanning mode with excitation from the 488 nm line of an Argon laser. Images were collected in data sets of two channels (500–550 nm and 660–720 nm) for fresh- and glutaraldehyde-fixed cells or in three channels for Nile Red-treated cell suspensions (500–550 nm, 570–620 nm and 660–720 nm) and examined as maximum projections (8–12 micrometers deep) in separate and graphically overlaid image channels. Fluorescence emission scans were performed from 500–750 nm using a 15 nm detector window and frame averaging of selected focal planes. DIC images were taken using the same method for laser-scanning confocal microscopy; however, the transmitted light channel (non-confocal) was employed here.

### 5.4. Microplate Bioassays

A fluridone standard obtained from Sigma-Aldrich, Inc. (St. Louis, MO, USA) was dissolved in 100% ethanol and diluted accordingly: 304, 152, 76, 38, 19, 0 µM. *Chlorococcum* sp. culture was added to two individual 96 well microplates from BRAND^®^ GmbH & Co. KG (Wertheim, DE) at an optical density (OD) of 0.1, 250 µL per well; adapted from Franz, et al., 2013 [48]. Fluridone was subsequently added to the microplates according to the above concentrations and incubated for ten days under the following conditions: light (22 µE per s^−1^m^−2^), CO_2_ (1%), temperature (24 °C). Plates were read at wavelengths 750 nm (biomass), 680 nm (chlorophyll), and 450 nm (carotenoids) over a ten day period using a SPECTRAmax microplate spectrophotometer, Molecular Devices (Sunnyvale, CA, USA).

### 5.5. Scale-up Bioassays

*Chlorococcum* sp. was added to twelve sterile BD Falcon™ Tissue Culture Flasks with vented caps from BD Biosciences (Erembodegem, BE) at 25 mL per flask, OD 0.5. A total of six flasks were incubated with fluridone at 152 and 304 µM, respectively and subjected to the following two conditions: (1) light (22 µE per s^−1^m^−2^), CO_2_ (1%), temperature (24 °C); (2) high light (2,000 µE per s^−1^m^−2^), CO_2_ (1%), temperature (24 °C). Controls did not contain fluridone. Experiment was performed in biological replicates of three. UV spectrophotometric readings were taken daily on a SPECTRAmax microplate spectrophotometer, Molecular Devices (Sunnyvale, CA, USA), for a total of four days.

### 5.6. Carotenoid Analyses & Quantification

Phytoene Profile–Algae was collected by centrifugation at 10,000 rpm and lyophilized on a Labconco FreeZone 6 (Kansas City, MO, USA) for a minimum of 24h. Approximately 15 mg of algae was milled with 0.5 mm dia. zirconia/silica beads (BioSpec Products, Inc., Bartlesville, OK, USA) using a Mini Bead Beater™ (BioSpec Products, Inc., Bartlesville, OK, USA) for a total of 2 min to achieve cell lyses. HPLC grade acetone (Sigma-Aldrich Corp., St. Louis, MO, USA) was added to the samples in a 1:30, mg: μL, ratio and allowed to sit for 20 min., followed by centrifugation for 10 min at 13,000 rpm and the supernatant removed and collected in a separate vial. The extraction was repeated a second time to ensure complete pigment-tissue extraction, and the supernatants combined. Samples were analyzed on a Waters 2695 Alliance^®^ HPLC (Waters Corp., Milford, MA, USA) with a 996 photodiode array detector and YMC America carotenoid column (YMC America, Inc., Allentown, PA, USA), using the YMC MTBE carotenoid method [54]. Phytoene quantification was performed using external calibration on a series of dilutions of a phytoene standard obtained from Sigma-Aldrich, product #78903 (Sigma-Aldrich Corp., St. Louis, MO, USA). Carotenoid content was quantified via UV-Vis using a series of dilutions of a β-carotene standard obtained from Sigma-Aldrich, product #1065480 (Sigma-Aldrich Corp., St. Louis, MO, USA), and read at 450 nm using a SPECTRAmax microplate spectrophotometer, Molecular Devices (Sunnyvale, CA, USA).

### 5.7. Lipid Analysis

Fatty Acid Methyl Ester (FAME) profiles were obtained for each treatment group via base catalyzed transesterification. 2 mL of KOH in methanol (2N) was applied to dried, ground tissue (~5 mg), vortexed and incubated at ~37 °C for 30 min. Samples were allowed to cool for approximately 15 min. 1 mL of acetic acid (1M) was added to samples to quench the reaction. Subsequently, 2 mL of HPLC grade hexane with C23:0 ISTD at 50 ppm was added to samples and vortexed thoroughly. All reagents for FAME extraction were obtained from (Sigma-Aldrich, St. Louis, MO, USA). 200 μL of the upper portion of the sample was removed and dispensed into GC vials, fitted with inserts, for analysis. Samples were then analyzed by GC/MS on a Varian 3800 Gas Chromatograph with a Varian 2000 Mass Spectrometer and a Varian 8200 Auto sampler (Agilent Technologies, Inc., Santa Clara, CA, USA). 2 μL were injected onto a 30 m × 0.25 mm diam. ×0.25 µm film DB-23 capillary column (Agilent Technologies, Inc., Santa Clara, CA, USA) with Helium carrier gas at 1 mL/min with a 5:1 split. The inlet and transfer line were held at 250 °C. The column temperature was held at 60 °C for 1 min. and then ramped at 30 °C min^−1^ to 175 °C and maintained for 1 min., then ramped to 235 °C at 4 °C for a total run time of 21.83 min. The instrument was tuned with a standard auto tune method and a calibration curve prepared from a Supelco 37 Component FAME mix (10 mg mL^−1^) in methylene chloride product # CRM47885 (Sigma-Aldrich, St. Louis, MO, USA). The mass spectrometer operated at 70 eV in electron ionization (EI) mode with 5 scans per second between the mass range 40 and 500.

### 5.8. Statistics

Statistics were performed using Sigma Plot V11.0 (Systat Software, Inc., San Jose, CA, USA). All data sets were run as a two-way analysis of variance (ANOVA), and using the Holm-Sidak method. Microplate bioassay data was run as a two-way ANOVA with repeated measures.

## Figures and Tables

**Table 1:** Total phytoene and carotenoids in *Chlorococcum* sp. with fluridone at 304 µM and 152 µM. n = 3, N.D. = not detected, SEM = standard error of the means.

	Total Phytoene (μg/mg)			Total Carotenoids (μg/mg)		
**Day 4 Harvest**						
**Treatments (μM)**	**MEAN**		**SEM**	**MEAN**		**SEM**
304	33.8	±	1.7	40.4	±	11.5
152	33	±	0.3	38.6	±	1.1
0	N.D.	±	N.D.	70.1	±	7.5
**Day 9 Harvest**						
**Treatments (μM)**	**MEAN**		**SEM**	**MEAN**		**SEM**
304	4.6	±	0.9	68.3	±	4.9
152	14.6	±	0.6	66.1	±	4.8
0	N.D.	±	N.D.	145.1	±	7.2

## Supplementary Materials

The following are available online at https://www.mdpi.com/1660-3397/17/3/187/s1, Figure S1: PCR amplification, Figure S2: Scanning Electron Microscopic images of *Chlorococcum* sp. (UTEX B 3056).

## Abbreviations

DOE	Department of Energy
GGPP	Geranylgeranyl pyrophosphate
PDS	Phytoene desaturase
PCR	Polymerase chain reaction
HPLC	High pressure liquid chromatography
MTBE	Methyl tert-butyl ether
SEM	Standard error of the means
FAME	Fatty acid methyl ester
FA	Fatty acid
DIC	Differential interference contrast
SEM	Scanning electron microscopy
ANOVA	Analysis of variance
