# Supplementary material for: Phytoene Accumulation in the Novel Microalga Chlorococcum sp. Using the Pigment Synthesis Inhibitor Fluridone
Source: Mar Drugs. 2019 Mar 22;17(3):187. doi: 10.3390/md17030187 (PMC6471924; doi:10.3390/md17030187)
Supplement: Supplementary file 1 [file marinedrugs-17-00187-s001.pdf]

Supplementary Material

Supplementary Figure 1 (S1). PCR amplification of 18S rDNA and RuBisCO large subunit from *Chlorococcum* sp. Lane one—1 kB marker; lane 2—blank, lane 3—DOE 0101 amplified 18S rDNA (725 bp), lane 4—blank, lane 5—DOE 0101 amplified RuBisCO large subunit (500 bp)

Supplementary Figure 2 (S2). SEM of *Chlorococcum sp.* DOE 0101 (**A**) single cell (**B**) cluster of cells with sporangia containing zoospores (**C**) vegetative cells (**D**) vegetative cells undergoing carotenogenesis

Supplementary Table 2 (S3). Total FAME concentration in *Chlorococcum* sp. with fluridone at 304 μM and 152 μM. n = 3 for all samples; excluding C16:2, where n = 2.

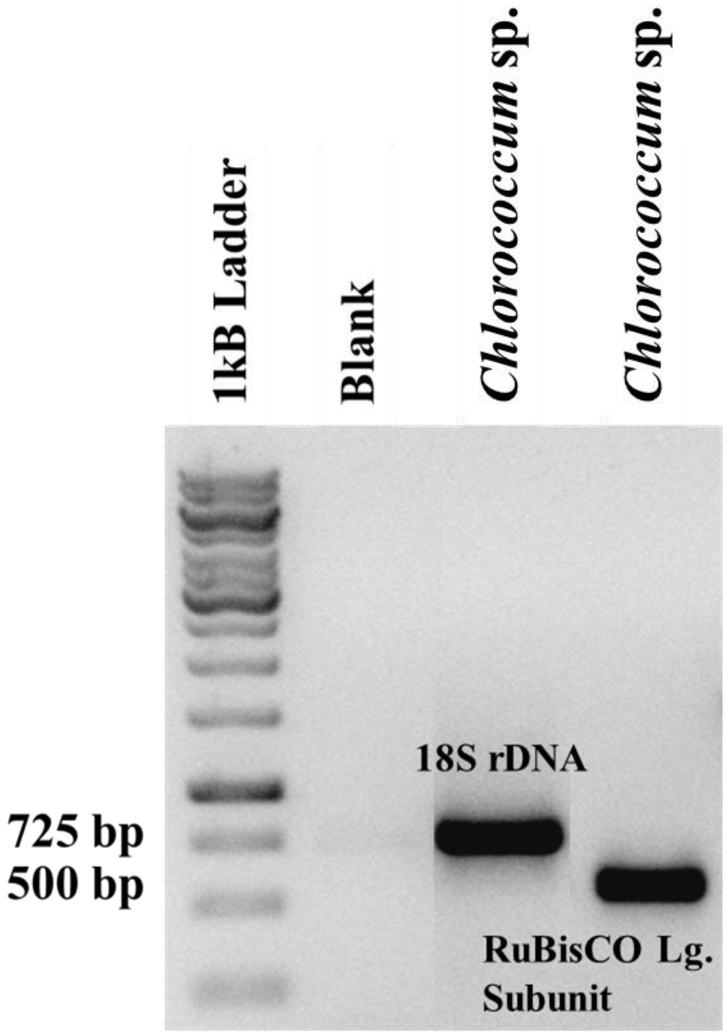

Supplementary Figure 1 (S1). Strain Identification—PCR amplification of 18S rDNA and RuBisCO large subunit from DOE 0101. Lane one—1 kB marker; lane 2—blank, lane 3—DOE 0101 amplified 18S rDNA (725 bp), lane 4—blank, lane 5—DOE 0101 amplified RuBisCO large subunit (500 bp).

Strain Identification

Molecular characterization and identification were performed on the genomic DNA extracted from *Chlorococcum sp.* DOE 0101 using the PowerSoil DNA Isolation Kit (Mo Bio Laboratories; Carlsbad, CA, USA). Regions of the 18S rDNA and the RuBisCo Large subunit were amplified from genomic DNA by polymerase chain reaction (PCR) using universal primer sets 18S rDNA (Forward—GTCAGAGGTGAAATTCTTGGATTTA, Reverse—AAGGGCAGGGACGTAATCAACG) and the RuBisCo Large subunit (Forward—AACCTTTCATGCGTTGGAGAGA, Reverse—CCTGCATGAATACCACCAGAAGC) and the GoTaq ® colorless master mix (Promega; Madison, WI, USA) according to the manufacturer's instructions. The PCR reactions were performed on a Mastercycler gradient machine (Eppendorf, Wesbury, NY, USA). The PCR program consisted of an initial denaturation/activation step at 95 ºC (3 min), 35 cycles of amplification [DNA denaturation step at 95 ºC (30 s), followed by an annealing step at 57 ºC (30 s) and an elongation step at 72 ºC (45 s)], and a final elongation step at 57 ºC (10 min). Amplicons were checked for size verification and specificity by gel electrophoresis on a 1% agarose gel. The amplicons were purified from gels using an UltraClean GelSpin® DNA extraction kit (Mo Bio Laboratories; Carlsbad, CA, USA) for subsequent forward and reverse sequencing (Sanger; ABI 3730 DNA analyzer) at the Functional Biosciences laboratory (Madison, WI, USA). Sequence data was analyzed and assembled using Geneious® (V6.1.4; Biomatters Inc.., Newark, NJ, USA). The consensus sequences were subjected to standard nucleotide similarity searches via BLASTn (Altschul et al., 1997) against the NCBI non-redundant database using standard parameters to determine their identities and assess their similarities to those in NCBI GenBank.

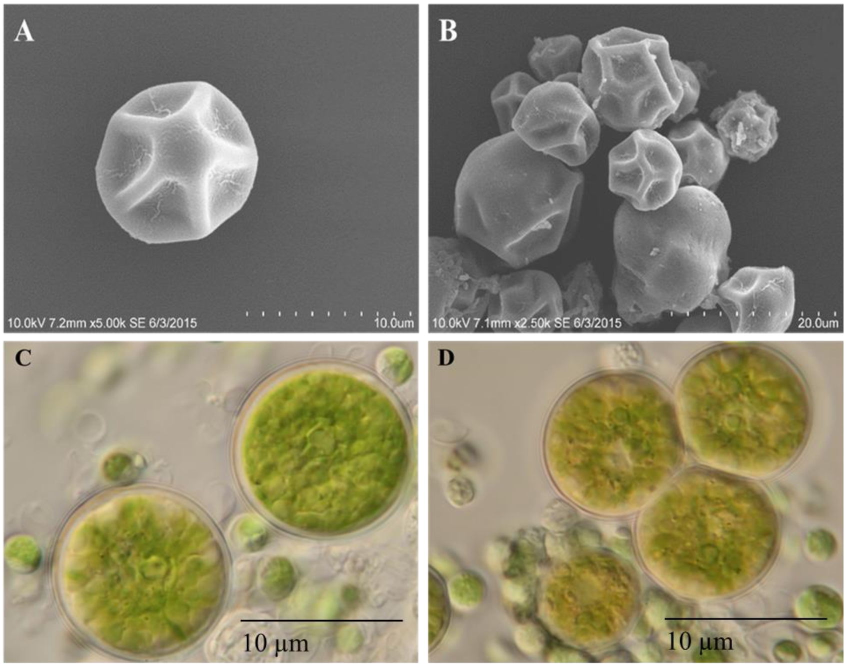

Supplementary Figure 2 (S2). SEM of *Chlorococcum sp.* DOE 0101 (**A**) single cell (**B**) cluster of cells with sporangia containing zoospores (**C**) vegetative cells (**D**) vegetative cells undergoing carotenogenesis.

Scanning Electron Microscopy

Morphological characteristics of the microalgae were captured using Scanning Electron Microscopy (SEM). Approximately 50 uL aliquots of concentrated suspensions of cultured cells were deposited on pre-cleaned (sonicated in acetone then absolute ethanol and finally air-dried) 12 mm dia. glass coverslips for 30–60 s before gentle immersion into a 2 mL volume of 2.5% glutaraldehyde (Electron Microscopy Sciences, Hatfield, PA, USA) buffered with 0.1 M imidazole-HCl at pH 7.2 in 24 microwell plates (CytoOne 24-well-non-treated-plate, USA Scientific, Inc., Ocala, FL, USA) and sealed overnight. Subsequently, the fixative solution was aspirated with transfer pipettes and replaced with the buffer solution for 20–30 min. Before dehydration in a graded series of ethanol solutions (50%, 80%, absolute) followed by 1:1 (v/v) solution of absolute ethanol and hexamethyldisilazane (HMDS) (ReagentPlus®, product # 379212, Sigma-Aldrich, Inc., St. Louis, MO, USA) for 20–30 min, followed by several changes of HMDS alone before air-drying from a small volume for approximately 15 h. Dry samples of cells adhering to the glass coverslips were mounted on aluminum sample stubs using carbon adhesive tabs and colloidal silver paint (Electron Microscopy Sciences, Hatfield, PA, USA) and finally examined in the high vacuum-secondary electron imaging mode of a model S-3400N scanning electron microscope (Hitachi High Technologies, Dallas, TX). Digital images were collected for characterization of the cellular topography.

Bright Field Microscopy

Bright field images of *Chlorococcum sp.* DOE 0101 were taken using a Zeiss Axioplan (Oberkochen, DE). Approximately 10 uL aliquots of concentrated suspensions of cultured cells were deposited on a glass slide and a coverglass was placed on top. Images were captured using a Canon Rebel T3 DSLR (Melville, NY, USA) mounted in the microscope camera mount using a Varimag II DSLR (CNC Supply, Inc., Cape Coral, FL, USA) and the 100× oil immersion objective lens.

Supplementary Table 2 (S3). Total FAME concentration in *Chlorococcum* sp. with fluridone at 304 μM and 152 μM. n = 3 for all samples; excluding C16:2, where n = 2.

Total FAME (μg/mg)									
Fatty Acid									
	C16:0			C16:1			C16:2		
Treatments (μM)	MEAN	±	SEM	MEAN	±	SEM	MEAN	±	SEM
304	3.87	±	0.07	11.84	±	0.01	10.87	±	0.01
152	3.75	±	0.07	11.96	±	0.16	10.6	±	0.56
0	4	±	0.06	11.46	±	0.18	8.083	±	0.23
	C16:3			C18:0			C18:1 cis		
Treatments (μM)	MEAN	±	SEM	MEAN	±	SEM	MEAN	±	SEM
304	12.16	±	0.01	1.58	±	0	2.37	±	0
152	12.11	±	0.01	2.41	±	0.04	2.41	±	0.04
0	12.06	±	0.05	2.37	±	0	2.5	±	0.06
	C18:1 trans			C18:2 cis			C18:3		
Treatments (μM)	MEAN	±	SEM	MEAN	±	SEM	MEAN	±	SEM
304	1.29	±	0.04	1.62	±	0	1.29	±	0.04
152	1.33	±	0.04	1.58	±	0.08	1.29	±	0.11
0	1.41	±	0.03	1.85	±	0.05	1.29	±	0.03

Fatty Acid Analysis

Fatty Acid Methyl Ester (FAME) profiles were obtained for each treatment group via base catalyzed transesterification. 2 mL of KOH in methanol (2N) was applied to dried, ground tissue (~ 5 mg), vortexed and incubated at ~37 ºC for 30 min. Samples were allowed to cool for approximately 15 min. 1 mL of acetic acid (1 M) was added to samples to quench the reaction. Subsequently, 2 mL of HPLC grade hexane with C23:0 ISTD at 50 ppm was added to samples and vortexed thoroughly. All reagents for FAME extraction were obtained from (Sigma-Aldrich, St. Louis, MO, USA). 200 μL of the upper portion of the sample was removed and dispensed into GC vials, fitted with inserts, for analysis. Samples were then analyzed by GC/MS on a Varian 3800 Gas Chromatograph with a Varian 2000 Mass Spectrometer and a Varian 8200 Auto sampler (Agilent Technologies, Inc., Santa Clara, CA, USA). 2 μL were injected onto a 30 m × 0.25 mm diam. ×0.25 μm film DB-23 capillary column (Agilent Technologies, Inc., Santa Clara, CA, USA) with Helium carrier gas at 1 mL/min with a 5:1 split. The inlet and transfer line were held at 250 ºC. The column temperature was held at 60 º C for 1 min. and then ramped at 30 ºC min^{-1} to 175 ºC and maintained for 1 min., then ramped to 235 ºC at 4 ºC for a total run time of 21.83 min. The instrument was tuned with a standard auto tune method and a calibration curve prepared from a Supelco 37 Component FAME mix (10 mg mL^{-1}) in methylene chloride (Sigma-Aldrich, St. Louis, MO, USA). The mass spectrometer operated at 70 eV in electron ionization (EI) mode with 5 scans per second between the mass range 40 and 500.
